# Cancer Cachexia: Signaling and Transcriptional Regulation of Muscle Catabolic Genes

**DOI:** 10.3390/cancers14174258

**Published:** 2022-08-31

**Authors:** Vinay Kumar Rao, Dipanwita Das, Reshma Taneja

**Affiliations:** 1 Department of Medical Genetics, JSS Medical College, JSS Academy of Higher Education and Research, Mysuru 570015, India; 2 Department of Physiology, Healthy Longevity Translational Research Program, Yong Loo Lin School of Medicine, National University of Singapore, Singapore 117593, Singapore

**Keywords:** cancer, cytokine signaling, muscle wasting, transcription factors, epigenetics

## Abstract

**Simple Summary:**

An uncontrollable loss in the skeletal muscle of cancer patients which leads to a significant reduction in body weight is clinically referred to as cancer cachexia (CC). While factors derived from the tumor environment which trigger various signaling pathways have been identified, not much progress has been made clinically to effectively prevent muscle loss. Deeper insights into the transcriptional and epigenetic regulation of muscle catabolic genes may shed light on key regulators which can be targeted to develop new therapeutic avenues.

**Abstract:**

Cancer cachexia (CC) is a multifactorial syndrome characterized by a significant reduction in body weight that is predominantly caused by the loss of skeletal muscle and adipose tissue. Although the ill effects of cachexia are well known, the condition has been largely overlooked, in part due to its complex etiology, heterogeneity in mediators, and the involvement of diverse signaling pathways. For a long time, inflammatory factors have been the focus when developing therapeutics for the treatment of CC. Despite promising pre-clinical results, they have not yet advanced to the clinic. Developing new therapies requires a comprehensive understanding of how deregulated signaling leads to catabolic gene expression that underlies muscle wasting. Here, we review CC-associated signaling pathways and the transcriptional cascade triggered by inflammatory cytokines. Further, we highlight epigenetic factors involved in the transcription of catabolic genes in muscle wasting. We conclude with reflections on the directions that might pave the way for new therapeutic approaches to treat CC.

## 1. Introduction

The word cachexia comes from the Greek word *Kakos hexis* meaning ‘bad condition’. Cachexia is a complex syndrome which manifests at late stages of several chronic diseases including Cancer, Chronic Kidney Disease (CKD), Chronic Obstructive Pulmonary Disease (COPD), AIDS, etc. [1]. Cancer Cachexia (CC) is often recognized or assessed only when cancer patients show signs of a significant loss in body weight. It affects up to 80% of cancer patients at late stages and occurs earlier in patients with gastrointestinal and lung cancer. Indeed, 20–30% of cachectic tumor patients die due to CC rather than from the tumor itself [2]. Despite being recognized as a devastating cancer-associated condition, cachexia remains an unmet medical need. This is partly due to the complexity of the syndrome and partly to the lack of proper guidelines to define and diagnose the condition. In 2011, Fearon et al. proposed a framework to define and classify CC as a multifactorial syndrome with an ongoing loss of skeletal muscle mass with or without loss of fat mass, which cannot be reversed by nutritional support. A loss in body weight of >5% over 6 months is classified as cachexia [3]. While sarcopenia refers to the gradual age-related loss in muscle mass/strength, cachexia is associated with skeletal muscle loss due to illness/disease. In addition to dealing with the tumor itself, cancer patients suffer multiple problems including weight loss, anorexia, increased resting energy expenditure, metabolic changes and systemic inflammation [4]. All these symptoms contribute to a reduced tolerance to cancer treatment, poor quality of life and increased mortality in patients. 

## 2. Mediators of Skeletal Muscle Wasting 

In general, most cancer patients suffer from anorexia (loss of appetite). Even sufficient nutritional support fails to reverse the progressive weight loss. The metabolic changes differ between anorexic and cachectic patients [1,5]. This indicates that reduced food intake alone is less likely to contribute to the complex muscle-wasting process. 

**Figure 1:**
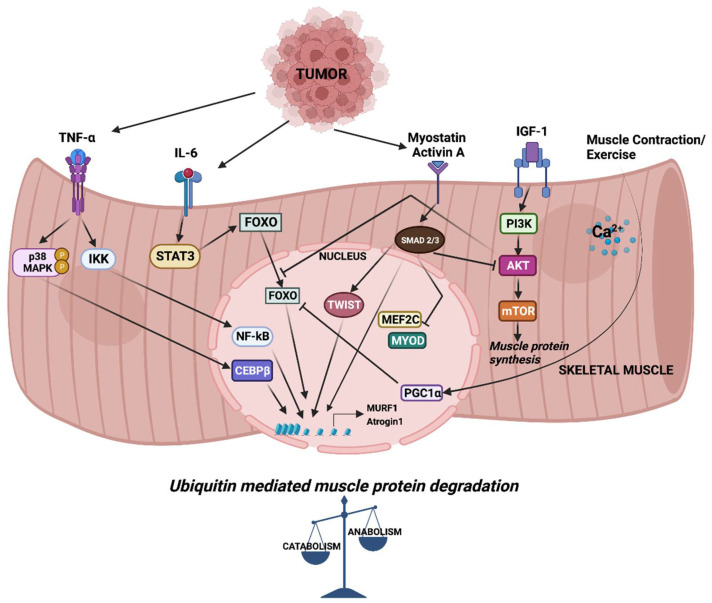
Signaling and downstream transcriptional response in skeletal muscle wasting. Cytokines released from tumor cells trigger signaling pathways which activate a cascade of transcription factors, leading to the expression of MURF1 and Atrogin-1. Increased expression of MURF1 and Atrogin-1 causes imbalance in muscle anabolic and catabolic processes.

Parabiosis studies in animal models provide evidence for the humoral mediation of CC [6,7]. These studies indicate that CC might be driven, at least in part, by circulating factors released by either host or tumor cells. Among these, inflammatory cytokines such as tumor necrosis factor alpha (TNFα), Interleukin-6 (IL-6) and transforming growth factor β (TGFβ) family members have been studied in detail. In CC, altered cytokine levels tilt the balance between muscle anabolic and catabolic genes towards catabolism, resulting in the degradation of muscle proteins. Cytokine-mediated signaling leading to the transcriptional regulation of muscle catabolic genes is critical in cachexia. In particular, the expression of the E3 ubiquitin ligase muscle RING finger containing protein 1 (MURF1) and muscle atrophy F box protein (MAFbx, also known as Atrogin-1) are central to ubiquitin-mediated myofibrillar protein degradation in cachectic skeletal muscle [8]. Several signaling pathways converge to activate the MURF1 and Atrogin-1 mediated degradation of skeletal muscle proteins that causes muscle wasting (Figure 1). Atrogin-1 controls the activity of the translation machinery protein eIF3f by ubiquitination [9]. Silencing Atrogin-1 leads to the upregulation of MyoD and the downregulation of myostatin, a negative regulator of muscle mass [10]. 

A large body of evidence consistently points to the involvement of the ubiquitin proteasome system (UPS) in the degradation of muscle proteins in CC. In humans, increased ubiquitin mRNA expression and increased proteolytic activity is evident in the muscle of gastric cancer cachexia patients [11,12]. A number of animal models of cachexia also showed increased activity of proteasomes [13]. Indeed, the inhibition of proteasome using MG132 in tumor-bearing mice leads to reduced cachexia [14]. 

## 3. Signaling Pathways and Transcriptional Mediators of CC

### 3.1. Phosphoinositide 3-Kinase (PI3K)-AKT Signaling

Under physiological conditions, the activation of PI3K-AKT downstream of Insulin-like growth factor 1 (IGF-1) signaling is critical for myogenic differentiation and an increase in skeletal muscle mass [15]. Reduced PI3K-AKT signaling causes muscle loss, while conversely, the activation of AKT leads to significant muscle hypertrophy in mice [16]. The IGF-1/PI3K/AKT axis promotes muscle growth through the mammalian target of rapamycin (mTOR) kinase and prevents the expression of MURF1and Atrogin-1 through the inhibition of Forkhead box protein O (FOXO) transcription factor, which controls the expression of the atrogenes involved in protein degradation [17]. FOXO transcription factors are predominantly involved in mediating the expression of E3 ubiquitin ligases during muscle atrophy. Myotubes undergoing atrophy show activation of FOXO transcription and an increased expression of Atrogin-1. Thus, the constitutive activation of FOXO3 increases Atrogin-1 expression and causes atrophy in myotubes and muscle fibers [18]. The expression of FOXO transcription factors is elevated in many CC mouse models, and its downregulation reduces muscle wasting. The inhibition of FOXO1 using specific RNA oligonucleotides results in increased skeletal muscle mass in cachectic mice [19]. Similarly, FOXP1 upregulation is sufficient to induce skeletal muscle wasting and weakness and is required for the normal wasting response in cancer. FOXP1 functions in a histone deacetylase (HDAC)-dependent mechanism and FOXP1 knockdown confers partial protection against cancer-induced muscle atrophy [20]. 

The transcription factor JunB, an activator protein-1 (AP-1) family member, is excluded from the nucleus in atrophying myotubes. When overexpressed, JunB induces muscle hypertrophy that is independent of the AKT/mTOR pathway. Moreover, the transfection of JunB into denervated muscles blocks the binding of FOXO3 to MURF1 and Atrogin-1 promoters, thereby reducing muscle protein breakdown [21]. Exercise-induced expression of the transcriptional co-activator peroxisome proliferator-activated receptor gamma coactivator 1alpha (PGC1α) counteracts FOXO-mediated muscle protein degradation. The expression of PGC1α in a mouse model of atrophy reduces the FOXO3-mediated expression of Atrogin-1 and muscle fiber atrophy [22]. 

Similar to IGF-1, stromal cell-derived factor 1 (SDF1)-CXCR4 signaling positively regulates skeletal muscle differentiation [23]. Several genes from the CXCR4 family, such as SDF1 and p21 activated kinase 1 (PAK1), are downregulated in the atrophying muscles of Yoshida hepatoma-bearing rodents. In the skeletal muscle of cancer patients, the expression of SDF1 and CXCR4 is inversely co-related to the expression of MURF1 and Atrogin-1. The overexpression of SDF1 or CXCR4 reduces muscle atrophy and increases muscle fiber diameter, implying that activation of the CXCR4 pathway may circumvent the muscle wasting seen in cancer [24]. 

### 3.2. TNFα and Nuclear Factor Kappa B (NF-κB) Signaling

In humans, serum TNFα is upregulated in hepatocellular cancer patients [25]. Moreover, TNFα levels in the serum of pancreatic and prostate cancer patients with metastasis are higher compared to non-metastatic patients [26]. Elevated TNFα expression is also evident in several animal models of cachexia [27,28]. In addition, the expression of the TNFα receptor adaptor protein, TRAF6, is increased during muscle atrophy. Several groups have shown the involvement of TNFα and NF-κB signaling in cachexia. TNFα, also known as cachectin, was first identified in a wasting condition associated with leishmaniasis in rabbits [29]. Later, it was shown to induce cachexia in mice [30]. TNFα inhibits skeletal muscle differentiation by promoting the proliferation of satellite cells through NF-κB signaling [31]. Along similar lines, TNFα-mediated NF-κB signaling suppresses MyoD transcription, causing the inhibition of skeletal muscle differentiation [32]. In pancreatic cancer patients and tumor-bearing mice, cachexia causes muscle damage, triggering the activation of satellite cells and non-satellite muscle progenitors which are present in the myofiber microenvironment. However, cells do not complete differentiation due to the NF-κB-induced persistent expression of paired box 7 (Pax7). In fact, the overexpression of Pax7 is sufficient to induce muscle wasting in mice, and its depletion in tumor-bearing mice reverses wasting [33]. These findings highlight the involvement of cells present in the muscle extrinsic microenvironment, i.e., outside the myofiber, in contributing to cachexia. In L6 myotubes, TNFα activates NF-κB and Forkhead transcription factor FKHR, leading to the induction of MURF1 and Atrogin-1. In addition, TNFα reduces PI3K-AKT signaling and leads to decreased myofiber diameter in L6 myotubes [34]. Loss of Sirtuin 1 (SIRT1) induces NF-κB signaling in cachectic muscles, activating the expression of FOXO and NADPH oxidase 4 (Nox4), which trigger protein degradation pathways in pancreatic cancer. The knockdown or pharmacological inhibition of Nox4 activity abolishes tumor-induced cachexia in mice, suggesting that targeting the SIRT1-Nox4 axis in muscles mitigates cachexia in pancreatic cancer [35]. High mobility group box protein B1 (HMGB1), which functions through the toll-like receptor 4 (TLR4)-NF-κB pathway, causes muscle atrophy through the upregulation of Atrogin-1 and MURF1. Either the downregulation of HMGB1 or the administration of its inhibitor glycyrrhizin reduces muscle protein degradation and alleviates muscle wasting in vivo [36]. 

Denervation combined with CC aggravates muscle atrophy in mice and induces the selective loss of myosin by both autophagy-mediated protein breakdown, as well as impaired protein synthesis [37]. TNF-like weak inducer of apoptosis (TWEAK) mediates skeletal muscle atrophy under denervation conditions in mice. Denervation-induced atrophy leads to the upregulation of TWEAK receptor fibroblast growth factor-inducible receptor 14 (Fn14), upregulating the expression of MURF1 through the NF-κB pathway [38]. Recent studies highlight that ER stress markers and unfolded protein response (UPR) pathways are activated in atrophic skeletal muscle in response to denervation, starvation and cancer [39]. TRAF6, a E3 ubiquitin ligase, mediates starvation-induced muscle atrophy in mice. The skeletal muscle-specific deletion of TRAF6 suppresses the expression of key atrophy genes including MURF-1 and Atrogin-1. Moreover, the expression of UPR genes such as ATF4, CCAAT/enhancer-binding protein homologous protein (CHOP), the ER stress-inducible enzyme PD1, and growth arrest and DNA damage-inducible protein (GADD34), were decreased in the skeletal muscle of TRAF6 knockout mice. This indicates that TRAF6 functions as a regulator of starvation-induced muscle atrophy [40]. Overall, these findings suggest that TNFα mediates muscle wasting via multiple pathways and, in particular, the TNFα-NF-κB axis plays a vital role as a mediator of muscle wasting in CC. 

Consequently, targeting TNFα to mitigate its ill effects in CC has been tested. The pre-emptive administration of TNFα blockers infliximab or adalimumab significantly inhibits TNFα and its downstream signaling in C26 cachectic mice. Compared to control cachectic mice, adalimumab-treated cachectic mice showed lesser weight loss, preservation of leg muscle and prolonged survival, along with the reduced expression of E3 ligases [41]. However, clinical trials using TNFα biologics such as etanercept, infliximab, and thalidomide have largely failed, indicating that additional signaling pathways may be involved in muscle wasting in cachexia [42,43,44]. 

Mitogen-activated protein kinases (MAPK) are serine/threonine protein kinases which include p38 MAPK, c-Jun N-terminal kinase (JNK) and extracellular signal-regulated kinase (ERKs). p38 MAPK signaling plays a critical role in myogenic differentiation. p38 kinases are recruited to muscle gene promoters and function in chromatin remodeling by targeting the SWI-SNF complex [45]. The p38-mediated activation of CCAAT/enhancer-binding protein β (C/EBPβ) has been extensively studied in the context of muscle wasting. A high expression of C/EBPβ is associated with tumor aggressiveness in cancers. Using both gain-of function and loss-of function approaches, C/EBPβ was shown to be sufficient to induce cachectic factors causing muscle atrophy and the inhibition of skeletal muscle differentiation [46]. Interestingly, the p300-mediated activation of C/EBPβ is required for cancer-induced cachexia. Either knockout or pharmacological inhibition of p300 abrogated muscle wasting in cachectic mice. Further, the p38β MAPK-mediated serine phosphorylation of p300 is critical for the acetylation of C/EBPβ, which mediates muscle wasting [47]. Similarly, the activation of p38β MAPK, but not p38α MAPK, was shown to be necessary and sufficient for Lewis lung carcinoma-induced autophagy, which leads to muscle wasting. p38β MAPK activated C/EBPβ causes the upregulation of autophagy-related genes LC3b and Gabarapl1. Thus, the muscle-specific knockout of p38β MAPK rescues muscle wasting in tumor-bearing mice [48]. The treatment of cachectic mice and atrophied myotubes with valproic acid (VPA), a HDAC inhibitor, showed reduced levels of C/EBPβ, which positively regulates Atrogin-1. VPA treatment caused a reduced interaction between HDAC and C/EBPβ, suggesting that targeting the HDAC-C/EBPβ interaction could be an approach for drug development [49]. These findings suggest that the modification of transcription factors including C/EBPβ by epigenetic factors plays a critical role in muscle wasting, and the prevention of the p38-mediated activation of p300 could be a strategy to treat cachexia. A transcriptome study involving the cachectic gastrocnemius muscle of a C26 xenograft model revealed DNA damage-inducible transcript 4 (Ddit4) as a key gene and mediator of muscle wasting in CC. Ddit4 expression is induced by p38, which inhibits the mTOR pathway in atrophic myotubes [50]. 

The aberrant activation of ERK signaling is associated with skeletal muscle wasting, and its inhibition prevents muscle wasting in the C26 cachexia model [51]. The treatment of C26 mice with selumetinib, a MEK/ERK pathway inhibitor, rescued the loss of body weight and attenuated expression of MURF1 and Atrogin-1. Selumetinib led to the activation of AKT and mTOR while inhibiting ERK, FOXO3a and GSK3β in cachectic mice [52]. Fibrinogen C domain-containing Protein 1 (Fibcd1), a secreted myokine, impedes the cancer-mediated upregulation of Ddit4. A recent study showed that Fibcd1, through ERK signaling, is responsible for myofiber size regulation in mice. Recombinant Fibcd1(rFibcd1) resulted in reduced myofiber atrophy associated with CC in the diaphragm. As rFibcd1 rescued muscle transcriptional changes induced by Lewis lung carcinoma cancers, interventions with rFibcd1 could be used to treat the loss of myofiber size in the diaphragm of cachexia patients [53]. 

The activation of JNK signaling, another MAPK family member, leads to reduced skeletal muscle differentiation [54]. The treatment of C2C12 cells with pancreatic cancer cell conditioned medium induces JNK signaling, causes myotube atrophy and increases the expression of Atrogin-1 and MURF1. Further, the inhibition of JNK signaling with SP600125 was shown to rescue loss of body weight and improve skeletal muscle strength by inhibiting the expression of atrogenes in an orthotopic pancreatic CC mouse model [55].

### 3.3. IL-6 and JAK/STAT Signaling

In humans, circulating IL-6 is found in non-small cell lung cancer patients. Higher IL-6 has a negative impact on patients’ survival [56,57]. Increased IL-6 is also reported in pancreatic and prostate cancer patients and is associated with poor performance, increased weight loss and higher mortality [58,59]. Increased IL-6 plasma levels result in impaired muscle growth during early postnatal development and induces muscle wasting [60]. Several studies have reported that the overexpression of IL-6 induces muscle loss in various animal models of cachexia [61,62]. In APC^Min/+^ mice, a genetic model of colon cancer, cachectic mice show higher circulating levels of IL-6, which is associated with loss in muscle oxidative capacity. A significant reduction in cytochrome-c and cytochrome-c oxidase complex subunit IV (Cox IV) is evident in cachectic mice [63]. In APC ^Min/+^ mice, IL-6 induces higher levels of Atrogin-1, exacerbating muscle loss. However, IL-6 overexpression does not induce cachexia in non-tumor-bearing mice [64]. The IL-6-induced increase in Atrogin-1 expression results in muscle growth deficits in mice, and this is reversed through voluntary endurance exercise [65]. 

IL-6 primarily mediates its effects through Janus kinase/Signal transducer and activator of transcription (JAK/STAT) pathway [66]. IL-6 is secreted in several cancers and may function independently of, or work in tandem with, TNFα to induce muscle wasting in cachexia. Myogenic differentiation involves the activation of STAT3 and its downstream target gene Socs3. The knockdown of IL-6 and STAT3 in C2C12 myoblasts leads to a reduced expression of myogenin and myosin heavy chain II b, causing impaired differentiation and the fusion of myoblasts. The knockdown of IL-6 alone does not prevent STAT3 phosphorylation, indicating the IL-6 independent activation of STAT3 in myoblasts. The study concluded that both IL-6 and STAT3 are required and necessary for myogenic differentiation [67]. 

Interestingly, IL-6 functions as a double-edged sword in regulating skeletal muscle cells. It stimulates muscle growth in physiological conditions and mediates muscle wasting in pathological conditions [68]. IL-6 has been identified as an essential regulator of satellite cell-mediated muscle hypertrophy. It is produced in myofibers and associated satellite cells, and the loss of IL-6 reduces the proliferation of satellite cells and blunts muscle hypertrophy in vivo. These findings highlight a context-dependent role for IL-6 in muscle growth [69]. The IL-6-mediated activation of STAT3 is a common feature in muscle wasting in both cell culture models, as well as in C26 cancer cachexia mouse model. The pharmacological inhibition of the STAT3 pathway blocks skeletal muscle wasting [70]. A recent finding showed that the STAT3/HSP90/FOXO1 axis plays a role in muscle wasting in cachexia. The inhibition of heat shock protein 90 (HSP90) reverses STAT3-mediated muscle wasting in vitro, as well as cachectic mouse models. The prolonged activation and binding of STAT3 results in the transactivation of FOXO1, triggering muscle wasting in a FOXO1-dependent manner in muscle cells [71]. 

In humans, phase I and II trials on cancer patients with ALD518, a humanized IL-6 antibody developed to treat cachexia was found to be safe and well tolerated [72]. Similarly, another monoclonal antibody for interleukin 1α, MABp1, was found to be safe, with no toxicities in phase I and phase III trials in colorectal cancer patients [73]. It is noteworthy that, similar to TNFα, targeting IL-6 alone may not be sufficient to treat or reverse skeletal muscle loss in cachexia. 

### 3.4. Suppressor of Mothers against Decapentaplegic (SMAD) Signaling

**Figure 2:**
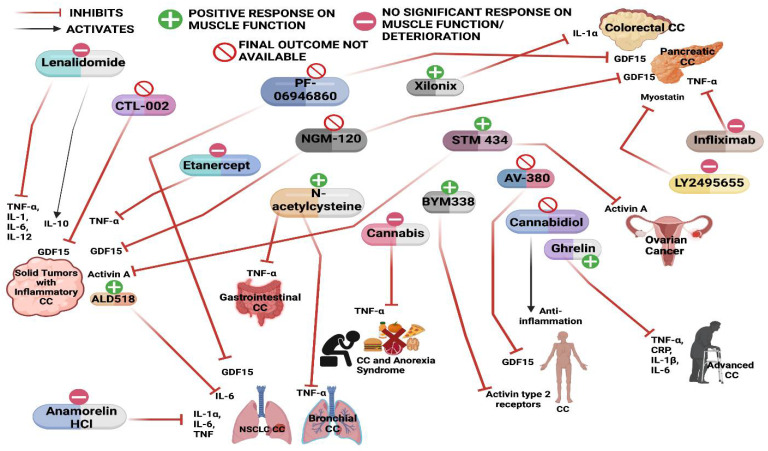
Schematic of various drugs used in the clinical trials for the treatment of cancer cachexia and their effect on muscle mass and function.

Myostatin is a TGFβ family protein expressed specifically in the myotome during early embryonic development and later in skeletal muscle tissue. Disruption of the myostatin gene in mice leads to increased skeletal muscle formation [74]. The overexpression of myostatin leads to the downregulation of MyoD expression, resulting in the inhibition of muscle differentiation [75]. The treatment of myotubes with myostatin inhibitor IMB0901 reverses the block of differentiation in atrophied C2C12 myotubes. IMB0901 treatment reduces the ubiquitin-mediated proteolysis of myogenic proteins and enhances AKT/mTOR- mediated protein synthesis [76]. The systemic overexpression of myostatin in mice leads to skeletal muscle loss resembling human cachexia syndrome [77]. Treatment with anti-human myostatin antibody (PF-354) in a mouse model of CC, where mice were injected with Lewis lung carcinoma tumor cells, resulted in reduced muscle atrophy and improvement in skeletal muscle mass [78]. However, recent phase II trials using another anti-myostatin antibody LY2495655 did not show much clinical benefit and did not improve the survival of patients when compared to placebo [79] (Table 1, Figure 2).

Similar to myostatin, activin A, another TGFβ family member, is also known to play a negative role in skeletal muscle mass. Both myostatin and activin A signal through the activin receptor ActRIIB, leading to the phosphorylation and dimerization of SMAD transcription factors (SMAD2 and SMAD3), which translocate to the nucleus to regulate gene expression [90]. Activin A signaling promotes pancreatic cancer progression and contributes to cachexia [91,92]. Consistently, circulating levels of activin A are found in cancer patients with significant weight loss [93]. This suggests that circulating activin A could be used as a biomarker for CC. The activin A/MEF2C pathway is involved in muscle wasting in vivo. Activin A inhibits the expression of myosin heavy chain and reduces skeletal muscle mass through the downregulation of MEF2C expression and activity [94]. MEF2C is an upstream activator of myocilin (Myoc), a skeletal muscle hypertrophy-promoting protein. Myoc is significantly reduced in cachectic pancreatic cancer patients, which correlates with reduced MEF2C [95]. 

While myostatin and activin A signaling promote muscle atrophy, bone morphogenetic protein (BMP) signaling, another TGFβ family member, functions as a positive regulator of muscle mass. The inhibition of BMP- SMAD1/5/8 signaling causes muscle atrophy and abolishes the muscle hypertrophy seen in myostatin-null mice. The muscle-specific knockout of SMAD4 results in a significantly higher loss of skeletal muscle compared to control mice upon denervation-induced muscle atrophy. This indicates that the differential recruitment of SMAD4 (a shared factor in BMP and myostatin signaling) to either the BMP or the myostatin pathway is critical for the balance between muscle atrophy and hypertrophy. Furthermore, BMP-SMAD1/5/8-SMAD4 signaling negatively regulates the Fbxo30 gene, which encodes the muscle ubiquitin ligase of the SCF complex in atrophy-1 (MUSA-1), which is involved in muscle protein degradation in atrophying muscle [96]. An increase in BMP7 expression or the constitutive activation of type I BMP receptors leads to the phosphorylation of SMAD1/5 and the activation of mTOR signaling, causing skeletal muscle hypertrophy. The inhibition of SMAD1/5 phosphorylation aggravates denervation-induced atrophy through a HDAC4-myogenin dependent mechanism, whereas the increased activity of BMP-SMAD1/5 protects muscle from neurogenic muscle wasting [97]. 

The nerve–muscle interface, particularly neuromuscular junction (NMJs), is relevant in understanding the pathophysiology of cachexia. Both the denervation and dysfunction of NMJs share similar characteristics of muscle wasting in conditions such as sarcopenia [98,99]. Despite the loss of skeletal muscle and increased muscular atrophy, NMJs are structurally stable, and morphology is conserved in human cancer cachectic patients [100]. However, recent evidence indicates that perturbations in NMJs are evident in C26 tumor-bearing cachectic mice [101]. The BMP pathway plays a role in regulating the formation of NMJs, and perturbed BMP signaling is evident in cachexia. Inflammatory factors including activin A and IL-6 trigger the expression of BMP inhibitors such as Noggin, thereby blocking the BMP signaling in muscle that causes altered NMJ structures in CC. Increasing BMP signaling through pharmacological means prevents muscle wasting and preserves NMJs in tumor-bearing cachectic mice [102]. These findings suggest that activating BMP signaling could have therapeutic benefits in CC.

The pharmacological blockade of ActRIIB in cachexia models not only prevents muscle loss but reverses the prior loss of skeletal muscle. ActRIIB antagonism reduces ubiquitin-mediated proteolysis and inhibits SMAD2/3 signaling, while enhancing satellite cell proliferation and regeneration [103]. Similarly, antagonizing ActRIIB by using recombinant adeno-associated viral vectors that increase SMAD7 expression results in reduced muscle wasting in mouse models of cachexia. SMAD7 prevents the activation of SMAD2/3 and degrades the ActRIIB complex [104]. As a result, molecules antagonizing the binding of ActRIIB ligands are in clinical development. In humans, a phase I study involving STM 434, an activin inhibitor, was used to assess the safety, pharmacokinetics and preliminary efficacy in advanced solid tumor patients. STM 434 treatment was shown to lead to an increase in the total lean body mass and an improved 6-min walk test time in patients. However, no evidence of anti-tumor activity was observed. Nonetheless, it warrants further exploration of activin A inhibitors as metabolic modulators in the treatment of CC [89]. 

### 3.5. Growth Differentiation Factor 15 (GDF15)

GDF15 is a distant member of the TGFβ family which functions through glial derived neurotrophic factor (GDNF) receptor alpha (GFRAL) and is known to play roles in CC [105,106,107]. Recent findings show that the expression and serum levels of GDF15 are increased in several diseases under stress conditions. This leads to alterations in pathways controlling appetite, thus, causing anorexia or cachexia [108]. The treatment of cachectic mice with a monoclonal antibody 3P10, which targets GFRAL and inhibits RET signaling, leads to a reversal of excessive lipid oxidation and calorie-restricted CC in mice [109]. Circulating levels of GDF15 were found to be higher in cancer patients receiving platinum-based chemotherapy and were associated with weight loss. Treatment with Anti-GDF15 antibody mAB1 was shown to improve survival and attenuate anorexia in both mice and non-human primates [110]. Overall, these studies suggest that blocking a common receptor for TGFβ family members might be meaningful in treating muscle wasting in CC. As a consequence, several clinical trials are directed to target TGFβ family members including myostatin, activin A and GDF15, which functions through SMAD2/3 signaling [111] (Table 1, Figure 2).

## 4. Epigenetic Regulation of Muscle Catabolic Genes

**Figure 3:**
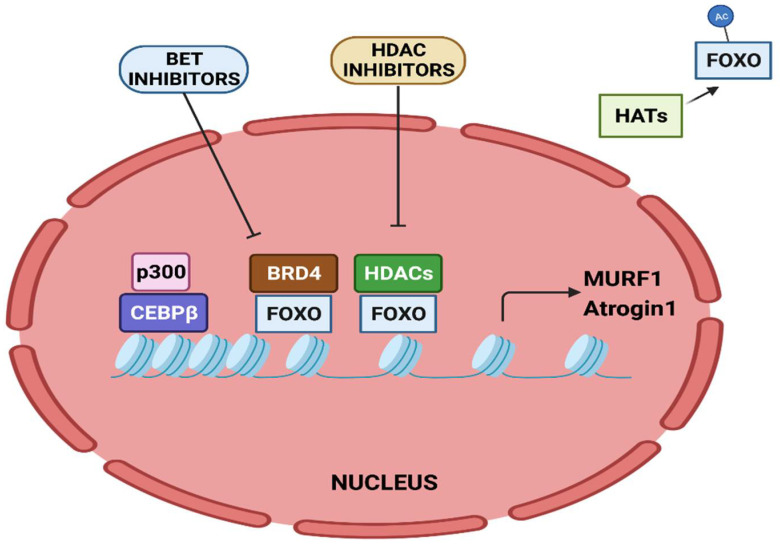
Epigenetic regulation of MURF1 and Atrogin-1 transcription. Transcriptional activation of MURF1 and Atrogin-1 by FOXO and CEBPβ is regulated by epigenetic factors.

The activity of FOXO transcription factors is tightly regulated by epigenetic factors (Figure 3). The acetylation of FOXO by p300-CBP acetyltransferase prevents nuclear localization and reduces its transcriptional activity [112]. HDAC1 increases FOXO activity, causing the elevated expression of atrogenes including MURF1 and Atrogin-1, and leads to muscle atrophy [113]. Trichostatin A (TSA), a HDAC inhibitor, counteracts unloading-induced muscle wasting. TSA treatment partly prevents the loss of type I and type IIa muscle fiber size in mice. Furthermore, HDAC4 interacts with and deacetylates FOXO3, leading to its activation, and thereby promotes denervation-induced muscle wasting in mice [114]. N^6^-methyladenosine (m^6^A) is one of the most abundantly studied post-transcriptional modifications of eukaryotic mRNA. Using a denervation-induced muscle atrophy mouse model, the m^6^A demethylase ALKB homologue 5 (ALKBH5) was shown to stabilize HDAC4 mRNA [115].

The bromodomain-containing BET protein promotes muscle wasting during cachexia. Administration of the BET inhibitor JQ1 in C26 tumor-bearing mice protects them from weight loss and muscle wasting. JQ1 administration orchestrates dual functions. It results in the loss of BRD4 at the promoters of catabolic genes. In addition, it results in reduced IL-6 levels, thereby restraining the IL-6/AMPK/FOXO3 activation of catabolic genes [116]. 

Twist1, a key transcription factor involved in epithelial to mesenchymal transition, is known to play a role in cachexia. Twist1 plays a critical role in muscle protein degradation in CC and its expression is higher in the skeletal muscles of cachectic mice models. Its expression is induced by activin A via SMAD signaling that activates Atrogin-1 and MURF1, causing muscle proteolysis. The conditional deletion or pharmacological inhibition of Twist1 suppresses muscle protein degradation in CC [117]. 

A loss in sarcomeric proteins, the contractile units of myofibrils, causes reduced muscle strength and induces wasting. SUMO-specific isopeptidase SENP3 determines sarcomere assembly by regulating the expression of sarcomeric contractile myosin heavy chain gene (MyHC-II). Under physiological conditions, SENP3 associates with the histone methyltransferase SETD7, causing its deSUMOylation. However, in cachectic muscle, SENP3 is degraded, which leads to the SUMOylation of SETD7. This allows the binding of SUV39H1 to MyHC-II, resulting in its repression, thereby causing disorganized sarcomeres [118]. Interestingly, chemotherapeutic drugs such as etoposide and daunorubicin are responsible for chemotherapy-induced cachexia. These drugs destabilize SENP3, causing disrupted sarcomere organization through the dissociation of SETD7 and acetyltransferase p300, which leads to reduced acetylation and the downregulation of sarcomeric genes. These findings highlight the role of epigenetic factors and SENP3-regulated mechanisms in cachexia [119]. 

Emerging evidence has demonstrated the association of non-coding RNAs with CC [120]. Novel miRNAs which are involved in myogenesis and metabolism have been identified in cancer cachexia [121]. Several miRNAs, including let-7d-3p, miR-345-5p, miR-532-5p, miR-378, miR-92a-3p and miR-21, are dysregulated in cachexia [122]. Integrated miRNA and mRNA co-profiles during skeletal muscle wasting in cancer-induced cachexia showed that extracellular matrix (ECM) associated genes are post-transcriptionally regulated by miRNAs (such as miR-29a-3p) and atrophy-related transcription factors including NF-κB, STAT3 and FOXO [123]. In addition, microarray data of long noncoding RNA (LncRNAs) in the adipose tissue showed that MALAT1 modulates adipose loss in CC by suppressing adipogenesis through PPAR-γ [124]. 

ANAPC7 circular RNA (circRNA) is a novel tumor suppressor in pancreatic cancer. It functions through the CREB–miR-373–PHLPP2 axis and leads to AKT dephosphorylation and the downregulation of TGFβ and cyclinD1 to suppress muscle wasting and cachexia. PHLPP2 induces the dephosphorylation of CREB, thus, regulating cancer progression and cachexia [125]. 

Tumor-secreted microvesicles contain an upregulated expression of miR-21, which induces myoblast apoptosis in CC through toll-like receptor 7/c-Jun N-terminal kinase-dependent pathway [126]. Poly ADP ribose polymerase (PARP), a muscle metabolic enzyme, plays a role in cancer-induced cachexia. When compared to WT cachectic mice, Parp 1^-/-^ and Parp 2^-/-^ lung cancer cachectic mice show improvement in muscle fiber size, muscle strength and reduced tumor burden [127].

Taken together, these studies highlight the interplay between epigenetic modifiers and transcription factors in regulating catabolic genes in muscle wasting. Given that CC does not involve somatic mutations, understanding the epigenetic regulation of catabolic genes might prove critical in the context of developing potential drug targets to treat muscle-wasting conditions. 

## 5. Treatment Approaches and Future Perspectives

Both the European Society of Medical Oncology (ESMO) and the American Society of Medical Oncology (ASMO) recommend a multimodal approach aimed at relieving symptoms of cachexia [128,129]. This includes food intake through nutritional support, ensuring adequate energy and nutrient intake, minimizing catabolic alterations through pharmacological intervention, supporting muscle training and offering psychological and social support to manage cancer cachexia. They emphasize the need to understand and treat the pathology from multiple angles. 

More than 40% of body mass is covered by skeletal muscle. The higher glucose consumption by cancer cells may result in muscle wasting as a natural response to sacrifice skeletal muscle and spare other critical organs for body function. The degradation of myofibrils, which are contractile units of muscle, leads to reduced muscle strength and mass. 

Recent studies show that circulating levels of activin A and GDF15 are similar between mouse models and human cancer patients, indicating that targeting these signaling networks could be beneficial [109,130]. Nevertheless, pre-clinical studies to target inflammatory cytokines and its associated pathways using inhibitors have not yielded promising results yet. This could partly be due to the differences in the animal models recapitulating human diseases and its associated conditions. It is becoming increasingly evident that one should look beyond inflammatory cytokine inhibitors to tackle skeletal muscle catabolism in cachexia. Activating muscle anabolic pathways including PI3K-AKT-mTOR signaling is a potential approach to increase muscle mass in cachexia. However, on the downside, AKT-IGF signaling can promote tumor growth and, hence, caution is warranted in its applications in cancer treatment. Its potential use is limited to non-cancer muscle-wasting conditions. 

Since cachexia involves differential gene expression with no underlying somatic mutations, epigenetics may play a crucial role in cancer cachexia pathology. Understanding the transcriptional response downstream of key signaling pathways may lead to the identification of potential targets for effective therapeutic interventions. For instance, BET inhibitors not only target factors such as FOXOs that control the expression of atrogenes, but also inhibit the expression of inflammatory cytokines such as IL-6. The activity of FOXO transcription factors is dependent on acetylation. Thus, targeting factors which are involved in deacetylation, such as HDACs, is an option. Similarly, specific inhibitors targeting p300 acetyltransferase activity could be effective in reducing C/EBPβ-dependent muscle wasting. Targeting miRNAs for therapeutic benefit to treat muscle wasting is yet another possibility. However, miRNA drugs are not yet being used in CC. miRNA-16 mimics are being tested in a phase-I clinical study for the treatment of non-small cell lung cancer [131]. miRNA mimics that have been used in clinical studies for cancer therapy can be evaluated for their potential in CC. Additionally, miRNA-based therapies targeting specific pathways for CC have the potential to restore homeostasis in chronically disrupted networks and positive muscle responses to exercise and diet. Studies have shown that the effectiveness of anticancer treatment has a direct impact on patients’ quality of life, as well as cachectic phenotype [132]. This reiterates that targeting epigenetic factors whose expression or function is altered in cancers could mediate cachexia. The identification of such factors could provide novel therapeutic avenues to treat muscle wasting in CC. 

## 6. Conclusions

It is apparent from the literature that despite continuous research and evolving knowledge, CC treatment remains a challenge due to its complexity and multifactorial mediators. The molecular and metabolic aberrations require a combination of both pharmacological and non-pharmacological interventions such as nutritional support and physical exercise. Improvements of CC treatment will need identification of key signaling and transcriptional nodes that regulate muscle catabolic genes that could lead to novel drug targets. 

## Figures and Tables

**Table 1:** Drugs in clinical trials for treatment of CC.

Drug /Stage of Trial/Clinical Trial Identifier	Condition/Disease	Mode of Action	Treatment Outcome
**Lenalidomide** [80] Phase-1 (Recruitment: Complete) NCT01127386	Solid tumours with inflammatory CC	Inhibits production of pro-inflammatory cytokines (TNF-α, IL-1, IL-6, IL-12) and elevates production of anti-inflammatory cytokines (IL-10).	No impact on muscle mass and muscle strength.
**Anamorelin HCl** [81] Phase-3 (Recruitment: Complete) NCT01395914	Non-small cell lung CC	Anamorelin is a novel, orally active, ghrelin receptor which reduces inflammation by inhibiting the expression of IL-1α, IL-6, and tumor necrosis factor.	Improved appetite, body weight and quality of life. However, overall survival and muscle function did not improve.
**Ghrelin** [82] Phase-1 Phase-2 (Recruitment: Complete) NCT00933361	Advanced CC	Ghrelin inhibits induction of pro-inflammatory cytokines such as TNF-α C-reactive protein, IL-1β, and IL-6, thus, regulating systemic inflammation in cancer patients.	Can enhance appetite and have anabolic effects.
**Cannabis Capsules** [83] (Recruitment: Complete) NCT02359123	Cancer-related cachexia and anorexia syndrome	Decrease in the level of pro-inflammatory cytokine TNF-α.	There was no significant difference in appetite, weight loss and quality of life. There was no observed toxicity.
**N-acetylcysteine** [84] Phase-2 (Recruitment: Complete) NCT00196885	Gastrointestinal/ Bronchial CC	Decreases the level of pro-inflammatory cytokine TNF-α.	Drug treatment strongly enhanced an increase in knee extensor strength.
**Cannabidiol** [85] Phase 1 (Recruitment: Active) NCT04585841	CC	Cannabidiol has anti-inflammatory properties, and prevents the imbalance between the pro- and anti-inflammatory cytokines.	Not available. (Estimated study completion: November 2022).
**PF-06946860** [86] Phase-1 (Recruitment: Complete) NCT04299048	Non-small-cell lung/pancreatic /colorectal CC	Inhibitor of growth differentiation factor-15 (GDF15), an atypical TGF-β superfamily member.	Not available.
**Infliximab** [87] Phase-2 (Recruitment: Complete) NCT00060502	Pancreatic CC	Infliximab is a monoclonal antibody that targets pro-inflammatory cytokine TNF-α.	Patients developed greater fatigue and a poorer quality of life score.
**ALD518** [88] Phase-2 (Recruitment: Complete) NCT00866970	Non-Small Cell Lung CC	ALD518 is a monoclonal antibody that targets IL-6 and represses inflammation mediated by IL-6.	There was attenuated loss of lean body mass and reversed fatigue in patients. There was significant difference in overall survival. Increase in levels of hemoglobin, hematocrit, and albumin seen in patients.
**Xilonix** [73] Phase-3 (Recruitment: Complete) NCT02138422	Colorectal cancer with cachexia	Xilonix is a monoclonal antibody that targets IL-1α and represses inflammation mediated by IL-1α.	Longer median overall survival and stable physical functions were observed in patients.
**Etanercept** [43] Phase-3 (Recruitment: Complete) NCT00046904	Solid tumours With inflammatory CC	Etanercept is a monoclonal antibody that targets pro-inflammatory cytokine TNF-α.	No significant differences in body weight change, appetite, median survival, and pathogen infection rates.
**BYM338** [88] Phase-2 (Recruitment: Complete) NCT01433263	CC	BYM338 is a human monoclonal antibody that blocks the activin type 2 receptors which belong to the TGF-beta receptor family.	Greater increases in thigh muscle volume, lean body mass, and physical activity were observed in patients treated with the drug. However, patients showed significant weight loss, indicating adverse effects on other wasting symptoms in cancer patients.
**AV-380 ** Phase-1 (Recruitment: Active; not recruiting) NCT04815551	CC	AV-380 is a monoclonal antibody which binds growth differentiation factor-15 (GDF15).	Not available.
**CTL-002** Phase-1/2 (Recruiting) NCT04725474	Solid tumors	CTL-002 is a monoclonal antibody which binds growth differentiation factor-15 (GDF15).	Not available. (Estimated study completion: May 2025).
**NGM-120** Phase-1/2 (Recruiting) NCT04068896	Advanced solid tumors and pancreatic cancers	NGM-120 is a monoclonal antibody which binds growth differentiation factor-15 (GDF15).	Not available. (Estimated study completion: January 2025).
**STM 434** [89] Phase-1 (Recruitment: Complete) NCT02262455	Ovarian cancer and other solid tumors	STM 434 is a soluble receptor ligand trap targeting activin A, a protein in the TGF-β family.	Increase in total lean body mass observed in patients.
**LY2495655** [79] Phase-2 (Recruitment: Complete) NCT01505530	Pancreatic CC	Decreases myostatin levels.	The patients showed increased fatigue, anorexia and earlier death. The trial was terminated due to the adverse effects.
